# Readiness of health facilities to deliver non-communicable diseases services in Kenya: a national cross-sectional survey

**DOI:** 10.1186/s12913-022-08364-w

**Published:** 2022-08-02

**Authors:** Rita Ammoun, Welcome Mkhululi Wami, Peter Otieno, Constance Schultsz, Catherine Kyobutungi, Gershim Asiki

**Affiliations:** 1 Faculty of Médicine, Limoges Université, 2 Rue du Docteur Marcland, 87025 LIMOGES CEDEX, France; 2 African Population and Health Research Center, 2nd Floor Manga Close, Off Kirawa Road, P.O. Box 10787 – 0100, Kitisuru, Nairobi, Kenya; 3 Amsterdam UMC, location University of Amsterdam, Department of Global Health, Amsterdam Institute for Global Health and Development, Meibergdreef 9 1105 AZ, Amsterdam, the Netherlands; 4 Department of Women’s and Children’s Health (KBH), Karolinka Institutet, Tomtebodavägen 18A, 171 77 Solna, Sweden

**Keywords:** Healthcare facilities, Non-communicable diseases, Service availability, Readiness

## Abstract

**Background:**

Non-communicable diseases (NCDs) account for an estimated 71% of all global deaths annually and nearly 80% of these deaths occur in low- and middle-income countries. This study aimed to assess the readiness of existing healthcare systems at different levels of health care in delivering NCDs management and prevention services in Kenya.

**Methods:**

A cross-sectional survey of 258 facilities was conducted between June 2019 and December 2020 using multistage sampling, examining facility readiness based on the availability of indicators such as equipment, diagnostic capacity, medicines and commodities, trained staff and guidelines for NCDs management. Readiness scores were calculated as the mean availability of tracer items expressed as a percentage and a cut-off threshold of ≥ 70% was used to classify facilities as “ready” to manage NCDs. Descriptive and bivariate analyses were performed to assess the readiness of facilities by type, level, and location settings. Logistic regressions were used to identify factors associated with the readiness of facilities to provide disease-specific services.

**Results:**

Of the surveyed facilities, 93.8% offered chronic respiratory disease (CRD) diagnosis and/or management services, 82.2% diabetes mellitus, 65.1% cardiovascular disease (CVD), and only 24.4% cervical cancer screening services. The mean readiness scores for diabetes mellitus (71%; 95% CI: 67–74) and CVD (69%; 95% CI: 66–72) were relatively high. Although CRD services were reportedly the most widely available, its mean readiness score was low (48%; 95% CI: 45–50). The majority of facilities offering cervical cancer services had all the necessary tracer items available to provide these services. Modeling results revealed that private facilities were more likely to be “ready” to offer NCDs services than public facilities. Similarly, hospitals were more likely “ready” to provide NCDs services than primary health facilities. These disparities in service readiness extended to the regional and urban/rural divide.

**Conclusions:**

Important gaps in the current readiness of facilities to manage NCDs in Kenya at different levels of health care were revealed, showing variations by disease and healthcare facility type. A collective approach is therefore needed to bridge the gap between resource availability and population healthcare needs.

**Supplementary Information:**

The online version contains supplementary material available at 10.1186/s12913-022-08364-w.

## Background

Non-communicable diseases (NCDs) constitute one of the major global public health challenges in the twenty-first century [1]. An estimated 41 million annual deaths are caused by NCDs globally, representing 71% of all deaths. Nearly 80% of these deaths occur in low- and middle-income countries (LMICs), with 9 million of these classified as premature deaths occurring below the age of 70 years [2]. Diabetes mellitus, cardiovascular disease (CVD), cancers, and chronic respiratory disease (CRD) constitute 73% of NCD burden, and they all share four common individual risk factors: harmful alcohol use, unhealthy diet, tobacco use and physical inactivity [2].

Kenya, like many other LMICs, is going through a transition from communicable to NCDs in terms of disease burden [3]. As an example, NCDs account for 31% of all deaths in Kenya, over 50% of total hospital admissions, and 55% of hospital deaths [4]. The contribution of NCDs to the total Disability-adjusted life years (DALYs) in Kenya increased from approximately 20% in 2004 to 25% in 2012, and the total deaths from 22% in 2004 to 31% in 2015 [5]. In the same way, the burden of NCDs has risen rapidly throughout sub-Saharan Africa (SSA) in the past few decades. According to estimates, the region’s DALYs burden from NCDs increased from 19 to 30% between 1990 and 2017 [6]. Several factors have been implicated in the rise in NCDs, including trade globalization, rapid unplanned urbanization, changes in nutrition, demographic changes such as population growth, and environmental factors like climate change and air pollution [7]. Even younger age groups and poorer communities are becoming increasingly vulnerable to these factors, which contribute to the increased burden of NCDs in LMICs [6–8].

The increasing burden of NCDs in LMICs, particularly SSA, is often not matched with an appropriate healthcare response as the current health systems were designed to offer response mainly to acute infectious diseases [9–11]. According to recent evaluations of national capacities to manage NCDs in Uganda, Ghana and Zambia, there were significant shortcomings in the delivery of NCD-related services [1, 12, 13]. There is a need to generate evidence to understand the gaps in NCD services in resource-limited settings and explore feasible solutions to improve the capacity of existing healthcare systems in delivering for NCD services [14]. The aim of this study was to assess the current readiness of healthcare facilities to provide management and prevention services for diabetes mellitus, CVD, CRD, and cervical cancer at different levels of health care in Kenya.

## Methods

### Study design and setting

A nationally representative cross-sectional survey involving randomly selected health facilities was conducted in Kenya between June 2019 and December 2020. The core health services in Kenya are delivered through a system of six levels, defined in four tiers of health care, namely: community, primary care, county referral, and national referral [15]. This system facilitates the establishment of health activities that advocate for the accessibility, affordability, and availability of NCD services at all levels of health care [16]. Level 1 is the lowest, at the community care unit, with no physical infrastructure present. It is the foundation of the service delivery system in the country. The majority of health services provided at the community level are non-facility based, including patient tracing, health promotion activities and disease prevention education. In this context, the basic NCD preventive interventions are expected to be provided at the lowest health care level. The primary health care service unit is comprised of level 2 (dispensaries and small clinics) and level 3 (health centers and small maternity clinics) facilities. It is expected that functional health facilities at this level will be able to provide a variety of services, including early detection of conditions, screening, and referral, in addition to the delivery of other basic health care services. Level 4 are sub-county hospitals and provide services to complement the primary health care level facilities. They allow for a more comprehensive service delivery package and serve as the primary referrals at the county level. They are also expected to be able to offer one or more specialized diagnostic and clinical laboratory services. Level 5 consists of county teaching and secondary referral hospitals providing a comprehensive wide range of health care service interventions. In addition, they also provide internship services for medical staff, conduct health research, and serve as training centers for paramedical staff. Lastly, level 6 facilities are the apex of the health care system in Kenya, comprised of national teaching and referral hospitals at the tertiary level. Facilities at this level provide highly specialized health services and complete the set of health care available in the country. They also provide advanced services such as sophisticated diagnostic, therapeutic and rehabilitation, or specific-disease management services. Furthermore, they conduct biomedical research and training for health care specialists, they also serve as internship/apprenticeship centers for specialists. The public sector provides 51% of health services in Kenya [17].

### Sample size calculations

A sample size of 301 health facilities was estimated using the formula commonly used for the Service Availability and Readiness Assessment (SARA) surveys that are nationally representative (32). Using inputs from a pilot study on health facility readiness to deliver CVD treatment and prevention conducted in Machakos and Nairobi counties of Kenya between 2016 – 2017, 40% of health facilities were estimated to deliver some aspects of chronic disease management. A 15% margin of error and a design effect of 1.2 as recommended for SARA surveys were assumed in the sample size calculations [18]. A non-response rate of 10% was also assumed based on the SARA implementation guide recommendations.

### Selection of health facilities

The Kenya Health Master Facility List of 2019, comprising of level 2 to 6 facilities was used as the sampling frame for this survey [15]. Level 1 facilities were not included in the sampling frame for the reason that they do not have a physical structure to assess the availability of specific tracer items. Initially, all level 5 and level 6 facilities in the country were targeted for potential inclusion in the survey. For facility levels 2 to 4, a multistage sampling method was used to select the health facilities. Kenya was first stratified into six geo-political regions: Nairobi, Central, Coast and North-Eastern, Eastern, Nyanza and Western, and Rift Valley. Then from each region, an independent two-stage sample was drawn. At the first stage, two counties (or sub-counties in the case of Nairobi) were selected in each region. The counties were sampled with probability proportional to size, with size being the total number of healthcare facilities in the respective county. At the second stage, health care facilities were sampled in each county. The healthcare facilities were stratified by level of health care and type of management (private or public) and then a stratified simple random sampling was used to select the health facilities. A conversant health professional from each facility was identified to respond to the survey questions. However, in some cases, especially at higher-level facilities, more than one respondent participated as some indicator tracer items being assessed were located in different departments. The following criteria had to be met for participation: (i) the healthcare professionals should have worked for at least one year in the facility with a good understanding of the facility’s capacity and chronic diseases related services provided by the facility; (ii) the healthcare professionals should have voluntarily been willing to participate in the study and able to provide information related to the management of NCDs.

### Data collection

A structured facility assessment questionnaire (Additional file 1) was used to collect data, which was adapted from the World Health Organization package of essential non-communicable diseases (WHO-PEN) interventions tool [19]. At each facility, one questionnaire was completed on the availability of indicator items specific to the diseases. Where feasible, direct observations were conducted to verify the interview responses. Prior to the main survey, a pilot study was conducted in Nairobi following the design. Its aims were: (i) to test and evaluate the instrument design, (ii) to assess the feasibility of the study in local settings, (iii) to affirm the validity and reliability of the instruments, and (iv) to familiarize the study data collection team with the study questionnaires and procedures. Data from health facilities eligible for the main study were collected electronically on tablets using SurveyCTO (Dobility, Inc. Cambridge, MA, USA), a digital data collection platform. In total, 258 (86%) of the 301 initially targeted health facilities were successfully sampled. This level of response is acceptable according to SARA methodology guidelines, which specify a response rate of 80% as reasonably acceptable level of response [18]. The other targeted facilities did not participate either due to security concerns, inaccessibility of the facility, voluntary non-consent, or the facility was no longer operational at the time of the study.

### Service availability and readiness indicator variables

Firstly, the disease-specific services (service availability) were evaluated by calculating the percentage proportion of facilities that provided diagnosis and/or management services for each condition separately. Next, the capacity of facilities to provide specific NCD services (service readiness) was assessed based on the availability of predefined tracer items for service domains: trained staff and guidelines, equipment, diagnostic capacity, and medicines and commodities (Additional file 2). The service readiness calculations did not include facilities that did not provide diagnosis and/or management services as per government guidelines for the reason that a facility could not be expected to be "ready" for a service that they do not provide. A mean availability score for each service domain was calculated based on the mean availability of tracer items, and service-specific readiness was calculated as an overall composite score based on the mean availability of tracer items across all the service domains, expressed as a percentage. This study modeled the main outcome as binary, based on the mean readiness scores of health facilities to deliver specific services for each disease separately. Those facilities with a service-specific readiness score of 70% or higher were considered to be "ready" or having the resource capacity to deliver NCD interventions, otherwise, they were deemed “unready”. The predictor variables included facility characteristics: level of health care (levels 2 to 6), type of managing authority (private *vs.* public), and location settings: urban versus rural and regions in Kenya.

### Sample weights

The sampling scheme was self-weighting within strata (region, county, healthcare level, and facility type), but the probability of being sampled differed between strata, hence sampling weights were computed for each stratum. The probability of facilities being sampled was calculated as the number of facilities in the sample divided by the total number of facilities in the stratum. Thus, weights were calculated as the inverse of the probability of being sampled and assigned to the tracer items indicator variables in the final dataset for analyses.

### Statistical analysis

Descriptive analyses were carried out for all indicator items using methods that are appropriate for complex multistage surveys [20]. Proportions were expressed as percentage frequencies, and if a variable was assessed using domains, mean weighted service-specific readiness scores were calculated along with 95% confidence intervals (95% CI). The differences in facility readiness scores between types, levels, urban–rural and regional geographic locations of health facilities were analyzed using estimation methods and visualized using Gardner-Altman plots [21, 22]. All confidence intervals in the mean difference comparisons were bias-corrected and based on 5000 accelerated bootstraps resamples [21]. Lastly, both univariate and multiple logistic regressions, weighted and adjusted for the survey design were used to identify factors that were associated with the binary readiness outcome of healthcare facilities to provide specific NCD services. Due to small sample size, cervical cancer was only analyzed descriptively and not included in any subsequent regression analysis. For each of the remaining three diseases assessed, separate models were fitted. Facility background characteristics were included as covariates in the modeling and no interactions were considered. The following categories for each of the facility characteristics were used as reference (ref) categories for comparisons purposes only: facility level (ref = “level 5/6”), facility type (ref = “public”), urban/rural setting (ref = “rural”) and region (ref = “Rift Valley”). In addition, when interpreting the results, emphasis was not only placed on significance, but also on relevance to the subject matter, therefore modeling results were reported as odds ratios (OR), both crude and adjusted, together with their corresponding 95% CI. The analyses were weighted to account for disproportionate sampling of facilities. Data analysis was carried out in SAS statistical software version 9.4 (SAS Institute, Cary, NC, USA), using the procedures for complex surveys [20, 23]. Estimation statistics and the Gardner-Altman plots were performed using R Statistical Software (version 4.1.0; R Foundation for Statistical Computing, Vienna, Austria).

## Results

### Characteristics of health facilities

Of the 258 surveyed facilities, the majority (67.8%; n = 175) were publicly managed. The survey consisted mostly of primary health facilities; for which 54.3% (n = 140) were level 2 and 34.1% (n = 88) were level 3 facilities. In addition, the sample included three of the five national referral hospitals (level 6) in the country. Furthermore, more than half of the facilities (53.5%; n = 138) were located in urban areas, with a proportionate distribution among the different regions (Table 1). Considering the small sample sizes of level 5 and level 6 facilities, data from them were combined and analyzed together.

**Table 1:** Percentage of facilities that offer non-communicable diseases services (*N* = 258)

		**Service Availability: diagnosis and/or management**			
**Background characteristics**	**Number of facilities** **(n)**	**Diabetes mellitus** **(%)**	**Cardiovascular** **disease (%)**	**Chronic** **respiratory** **disease** **(%)**	**Cervical cancer** **(%)**
***Facility level***					
Level 2	140	71.4	57.9	90.7	14.3
Level 3	88	94.3	65.9	96.6	25.0
Level 4	19	94.7	94.7	100.0	57.9
Level 5/6	11	100.0	100.0	100.0	90.9
***Facility type***					
Private	83	82.9	55.9	79.0	13.9
Public	175	69.0	62.2	91.5	18.1
***Facility setting***					
Rural	138	68.4	57.6	85.3	13.0
Urban	120	87.2	59.8	83.2	19.0
***Region***					
Central	39	77.4	39.8	63.5	8.3
Coast & North-Eastern	45	96.5	69.1	97.9	33.8
Eastern	45	83.1	84.3	94.6	6.8
Nairobi	36	81.7	60.8	80.4	5.1
Rift Valley	46	44.7	30.2	90.7	12.9
Western & Nyanza	47	76.3	77.7	97.2	36.1
**Overall**	**258**	**82.2**	**65.1**	**93.8**	**24.4**

### Availability of diagnosis and/or management services

Most facilities reported that they offered diagnosis and/or management services for CRD (93.8%; *n* = 242) across all levels of health care, type of facility, and location settings (Table 1). Overall, 82.2% (*n* = 212) of the surveyed facilities provided diabetes mellitus services. In addition, a higher proportion of private and urban facilities offered diabetes mellitus services than public and rural facilities, respectively. Cardiovascular disease services were available in 65.1% (*n* = 168) of the surveyed facilities and these services were mostly available in higher-level facilities. Cervical cancer screening services were available in only 24.4% (*n *= 63) of facilities, mostly offered in hospitals (level 4 and above). In addition, a smaller proportion of facilities in the Rift Valley reported that they provided diabetes mellitus and CVD services than other regions (Table 1).

### Facility service-specific readiness

**Fig. 1:**
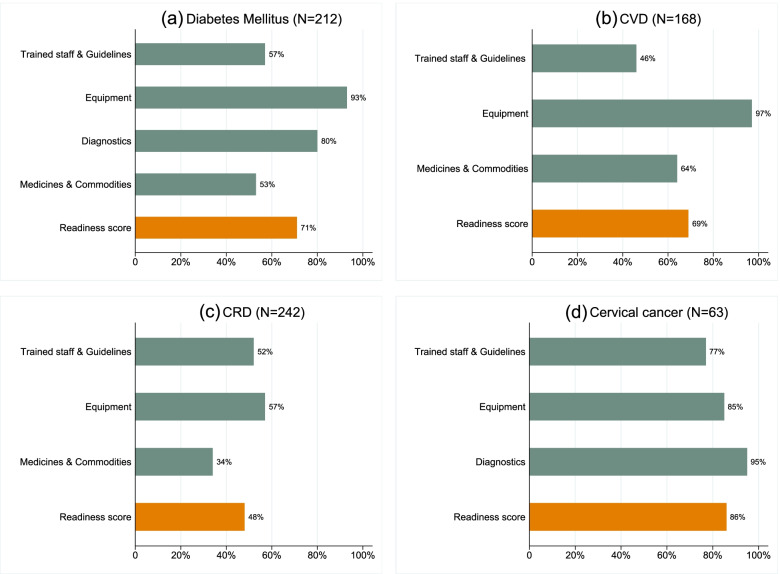
Service-specific percentage domains and mean readiness scores for facilities that offered non-communicable diseases services

Figure 1 shows the percentage service-specific domains and overall service-specific readiness scores for each of the diseases. The diabetes mellitus services mean readiness score was 71% (95% CI: 67 – 74), that is, on average facilities had approximately three quarters of the tracer items available. Of these facilities offering this service, 12% (*n* = 25) had all the necessary tracer items available. The availability of equipment and diagnostic test services for diabetes mellitus was high. The overall readiness score for CVD was relatively high (69%; 95% CI: 66 – 72). However, noticeable shortcomings in the availability of trained staff and national guidelines for this service were observed (Fig. 1). While CRD services were widely offered among facilities, the mean availability of tracer items was very low (48%, 95% CI: 45 – 50), compounded by the substantial lack of essential medicines and commodities (Fig. 1). The readiness for cervical cancer management among the few facilities offering this service was very high (86%, 95% CI: 82 – 89). In addition, most of the facilities offering this service had the necessary diagnostics (acetic acid) and equipment (speculum) essential for cervical cancer screening.

### Differences in facility service-specific readiness scores

**Fig. 2:**
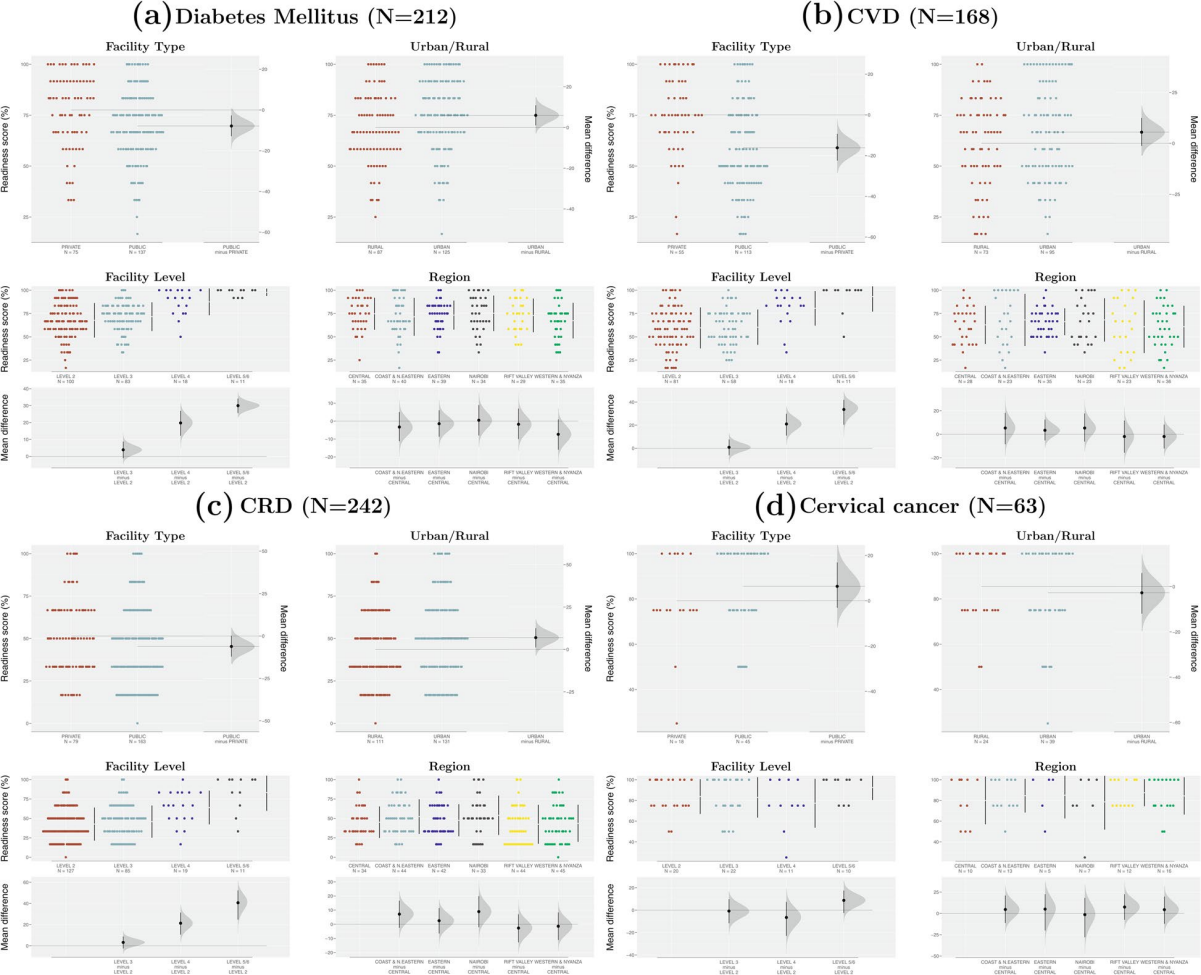
Comparisons of service-specific readiness scores by facility characteristics Gardner-Altman estimation plots for comparing mean service readiness scores, by facility level, type, urban/rural, and region: (**a**) diabetes mellitus; (**b**) cardiovascular disease (CVD); (**c**) chronic respiratory disease (CRD); (**d**) cervical cancer.

The distributions of facility service-specific readiness scores for each disease by background characteristics are shown in Fig. 2 (panels a to d). For all conditions except cervical cancer, there were apparent differences in service readiness scores by facility level, with notable differences observed between primary health care facilities at the lower-level (levels 2 and 3) and hospitals (higher-level facilities). In addition, variations in the distribution of facility service-specific readiness scores between private and public, as well as urban–rural health facilities were observed. Across all diseases, the magnitude of regional differences in facility service readiness scores was very marginal (Fig. 2).

### Assessing factors associated with facility service readiness

Table 2 shows the results of both the unadjusted (crude) and predictor-adjusted logistic regression models for each of the three diseases, separately. Adjusting for other facility background characteristics, the results showed that private (OR = 3.58; 95% CI: 2.92 – 4.40; *P* < 0.001) and urban (OR = 3.25; 95% CI: 2.62 – 4.03; *P* < 0.001) facilities were at least three times more likely to be “ready” to deliver diabetes mellitus-related services than public and rural facilities, respectively. Furthermore, variations by the level of health care in the readiness of facilities to provide diabetes mellitus services were also observed, with level 2 and 3 facilities less likely to be “ready” to provide diabetes mellitus services than higher level health care facilities. Disparities were also noted by region. Facilities in the Rift Valley, for example, were found to be less likely “ready” to provide diabetes mellitus services. These results were also true for other diseases assessed in this study.

**Table 2:** Factors associated with the readiness of facilities to provide non-communicable diseases management services

**Background characteristics**	**Diabetes mellitus service readiness (*N* = 212)**		**CVD service readiness (*N* = 168)**		**CRD service readiness (*N* = 242)**	
	**Unadjusted OR** **(95% CI)**	**Adjusted OR** **(95% CI)**	**Unadjusted OR** **(95% CI)**	**Adjusted OR** **(95% CI)**	**Unadjusted OR** **(95% CI)**	**Adjusted OR** **(95% CI)**
***Facility Level***						
Level 2	**0.03 (0.00 – 0.64)**	**0.03 (0 – 0.56)**	**0.02 (0.00 – 0.47)**	**0.01 (< 0.001 – 0.17)**	**0.04 (0.01 – 0.14)**	**0.05 (0.01 – 0.19)**
Level 3	**0.04 (0.00 – 0.72)**	**0.03 (0 – 0.57)**	**0.03 (0.00 – 0.61)**	**0.02 (< 0.001 – 0.44)**	**0.05 (0.02 – 0.18)**	**0.04 (0.01 – 0.15)**
Level 4	2.11 (0.09 – 48.03)	2.57 (0.11 – 61.89)	0.19 (0.01 – 3.72)	0.11 (0.01 – 2.40)	0.50 (0.15 – 1.69)	0.80 (0.21 – 3.06)
Level 5/6	(ref)	(ref)	(ref)	(ref)	(ref)	(ref)
***Facility Type***						
Private	**2.80 (2.36 – 3.32)**	**3.58 (2.92 – 4.40)**	**4.90 (4.00 – 5.99)**	**5.57 (4.35 – 7.14)**	**1.73 (1.34 – 2.23)**	1.16 (0.84 – 1.60)
Public	(ref)	(ref)	(ref)	(ref)	(ref)	(ref)
***Urban/Rural***						
Urban	**3.21 (2.72 – 3.79)**	**3.25 (2.62 – 4.03)**	**1.91 (1.58 – 2.30)**	1.22 (0.95 – 1.57)	**6.42 (4.74 – 8.69)**	**5.63 (3.90 – 8.12)**
Rural	(ref)	(ref)	(ref)	(ref)	(ref)	(ref)
***Region***						
Central	1.16 (0.83 – 1.60)	0.88 (0.60 – 1.29)	**3.90 (2.53 – 6.01)**	**6.80 (4.16 – 11.1)**	0.86 (0.43 – 1.73)	0.77 (0.35 – 1.67)
Coast & North-Eastern	**3.48 (2.43 – 4.97)**	**4.46 (2.96 – 6.73)**	**3.51 (2.25 – 5.47)**	**4.33 (2.63 – 7.12)**	**11.18 (6.35 – 19.68)**	**16.62 (8.64 – 32.00)**
Eastern	**1.36 (0.97 – 1.91)**	**2.12 (1.43 – 3.15)**	0.81 (0.53 – 1.25)	1.55 (0.96 – 2.50)	1.18 (0.61 – 2.29)	1.12 (0.53 – 2.36)
Nairobi	**3.95 (2.67 – 5.86)**	**2.01 (1.27 – 3.18)**	**3.75 (2.34 – 6.02)**	**3.77 (2.17 – 6.54)**	**5.08 (2.73 – 9.42)**	**3.15 (1.54 – 6.44)**
Western & Nyanza	1.36 (0.95 – 1.95)	1.36 (0.89 – 2.08)	**1.23 (0.8 – 1.91)**	**1.27 (0.78 – 2.07)**	**4.75 (2.65 – 8.54)**	**3.99 (2.04 – 7.83)**
Rift Valley	(ref)	(ref)	(ref)	(ref)	(ref)	(ref)

OR = Odds Ratio. Bold font indicates statistical significance

As with diabetes mellitus services, regression modeling results showed that facility type and level were associated with the readiness to provide CVD services (Table 2). However, the difference between urban and rural facilities in their readiness to provide CVD services was marginal and not significant (OR = 1.22; 95% CI: 0.95 – 1.57; *P* = 0.125), after adjusting for other facility characteristics.

In the univariate analysis, all facility background characteristics were associated with readiness to provide CRD-specific services (Table 2). The adjusted modeling results also showed that facilities in urban areas were more likely to be “ready” to provide CRD services than those in rural areas (OR = 5.63, 95% CI: 3.90 – 8.12; *P* < 0.001). The analysis, however, did not reveal significant variations in readiness by facility type.

## Discussion

The study investigated the current state of readiness of Kenyan health facilities to deliver NCD services. Three aspects of the NCD-specific services were evaluated; service availability, service readiness, and the assessment of factors associated with the readiness of health facilities to provide NCD services based on a 70% service-specific readiness score cut-off. Study findings highlighted both strengths and weaknesses in the existing health care system, as well as areas for improvement in regard to the management of NCDs. Firstly, the availability of diabetes mellitus, CVD, and CRD services was relatively good, with over two-thirds of health facilities reporting that these services were available. Diagnosis and/or management services for CRD were widely available at all levels of health care, types of facilities, and location settings. In contrast, only a quarter of the facilities surveyed provided cervical cancer screening.

Secondly, readiness to offer specific services for NCDs varied by disease, and important gaps were identified in the availability of tracer items at all levels, types of health care, urban versus rural facilities, and between the regions in Kenya. Diabetes mellitus was identified as the condition the majority of facilities were reasonably prepared to manage. Nonetheless, readiness was primarily influenced by the high availability of equipment tracer items rather than other service domain indicators. Similarly, although the overall CVD service readiness scores were relatively high, there were notable shortcomings in the availability of trained personnel and national guidelines on the management of this condition. Furthermore, the study revealed that even though CRD services were reported to be the most available in the facilities, the overall readiness to offer this service was generally poor and below the WHO recommended voluntary global target levels [24]. Nevertheless, facilities offering cervical cancer services were found to have high readiness scores for this condition because they had the necessary domain tracer items to provide the service. However, these results may not be a true reflection of the levels of readiness to manage this condition due to the small number of facilities assessed. As such, these results can only be viewed as descriptive.

Thirdly, the results of the logistic regression modeling revealed that private facilities were always more likely to be ready to provide NCD-specific services than public facilities. In addition, primary health care facilities were found to be associated with lower readiness to provide NCD management services than hospitals (at higher levels of health care). Furthermore, urban–rural differences in service-specific readiness were noted, particularly for diabetes mellitus services management, with urban facilities more likely to be “ready” to offer services for this condition than those located in rural areas. The modeling also revealed significant disparities in service readiness between regions. This pattern was observed for all the diseases assessed.

Our findings highlighting a lack of overall access to some essential specific NCD services are similar to reports from other previous studies, which concluded that NCD interventions were generally lacking in the country, particularly in the poorer regions and in the public sector [17, 25]. Nonetheless, there were also some positives to appreciate. The current study, for instance, found that the availability of equipment for NCD interventions was generally good, and this should be viewed as a positive step forward. In part, this could be attributed to the many policies and initiatives implemented by the Ministry of Health Kenya to reduce the burden of NCDs, including the Kenya National Strategy for the Prevention and Control of NCDs 2015–2020 [26].

Consistent with our current findings, previous studies have also shown disparities in the availability of health care resources for the prevention and control of NCDs between levels of health care, types of facilities, and their rural–urban locations [1, 24, 27]. These current findings are important in that they further highlight that where NCDs health services are needed most by the populations (that is, at primary health care, public and rural facilities), they are not always readily available in these settings. To further reiterate the message, it is important to identify and highlight these gaps in services because public and rural facilities are the most accessible units of health care for most populations in LIMCs. Thus, there is need for more resources need to be channelled towards these areas to close the gap and hence establish properly functioning health systems that are responsive to the health care needs for NCDs.

Closely related to the above findings, another cause for concern was the substantial lack of essential medicines and commodities for NCDs, particularly among the public and rural facilities consistently highlighted in our study and also reported in other previous studies [13, 28, 29]. Moreover, this was often accompanied by shortages of trained health professionals at these facilities. With limited access to medicines, the patient's best option may be to obtain them at higher-priced private facilities and private drug outlets [28]. This will more likely affect poorer members of the population such as those in rural areas, who are less likely to have the resource to travel long distances to access health care [9]. To further highlight this plight, a recent qualitative study on the perception of Kenyan adults on access to medicines for NCDs reported that when medications were not readily available, patients were more likely to only take a portion of the prescribed dose or even go for days without taking them at all [30]. This could potentially lead to disease progression and can also affect medication compliance when the treatment is considered taken yet the dosage is not adequate [30]. It is also important to emphasize that availability of medicines and health worker training domains complement each other, for instance, even if trained human resources were available to provide patients with services, the lack of essential medicines and commodities will prevent the health professional to deliver the appropriate health care, and vice versa [31]. There is therefore a compelling need to address medicines and trained staff shortages concurrently, as this could help improve management, consequently resulting in improved drug availability and supply.

These disparities in service-specific readiness have also been reported within countries or sub-regions of countries in SSA [29]. The widespread lack of essential resources has hindered progress in the management and prevention of NCDs in the region [32, 33]. For example, a study reviewing the progress of all 47 countries in the WHO African Regions revealed that none of the countries met all the recommended indicators for service-specific readiness [11]. Global trends have also highlighted similar gaps. A recent report by the WHO monitoring non-communicable disease progress in 2020 using data from 194 countries highlighted that the majority of countries, particularly LMICs, had not met the set global targets, further reiterating the urgent global need to advance work on NCDs prevention and control [2].

The findings of this study offer a reminder that has important implications for healthcare policy in the country. Furthermore, these current study implications also extend to other LMICs, as previously published evidence has highlighted the need for health systems strengthening and re-organization to ensure effective NCD prevention and control [34]. Despite CVD being the most common of the NCDs [35], facilities were less prepared to manage them than diabetes mellitus. There is therefore a need for services to be prioritized according to disease burden. The gaps identified for the different diseases at different types and levels of health care, as well as the notably regional and urban–rural disparities, coupled with sub-optimal availability of essential medicines and commodities, emphasize the need for a “complete package” approach to expanding the capacity of health facilities to deliver effective NCD interventions. Firstly, efforts need to be implemented at primary health care, as well as in public and rural facilities collectively, to ensure universal health coverage, since these facilities are more accessible to the majority of the population. This is relevant particularly when considering that patients with undiagnosed conditions who live in less prepared areas, and who may be asymptomatic for years are more likely to encounter inadequate screening, treatment, and referral to health care, resulting in long-term negative consequences such as chronic morbidity. Consequently, this could contribute to the rising disease burden and poor possible health outcomes from NCD interventions. These gaps could be addressed by re-prioritizing funds to provide these facilities with adequate diagnostic capacity, laboratory tests or procedures, and more importantly, by focusing on increasing accessibility to essential medicines at these facilities.

The variation of health facilities’ readiness between regions and urban–rural disparities should be also addressed. Kenya’s government, policymakers, and stakeholders must reconsider how resources are distributed to ensure equitable healthcare access. The findings highlighting disparities in terms of availability of trained staff and guidelines on NCDs are extremely important because poor knowledge and expertise of front-line healthcare professionals have already been identified as a major barrier to NCD health care in numerous studies in Sub-Saharan Africa [36]. These areas should be improved and addressed simultaneously as they have been shown to be cost-effective in terms of health care delivery [37]. Lastly, there is an urgent need to step up cervical cancer screening services to make them widely available as part of routine health care at all levels. A recent study revealed increasing trends in cervical cancer incidence in Sub-Saharan Africa, attributable to a lack of screening and prevention services [38].

### Strengths and weaknesses of the study

One of the strengths of this study is that it examined a sample of facilities spanning across all geographic regions of Kenya, ensuring national representativeness in terms of general health facilities characteristics within the country’s health system. A further strength was that the data were collected using an adapted WHO-PEN questionnaire, nevertheless, with a focus on NCDs. This tool is increasingly being used in other LMICs, allowing our results to be comparable with other studies. However, some limitations should be acknowledged when interpreting the findings of this study. Firstly, like any other survey as opposed to a census, there is an inherent risk of sampling bias. In the present study, steps were taken to ensure that the sampling design was robust and sample weights were applied to account for disproportionate selection of facilities. Another limitation was on those domain tracer items where the information could not be verified by visual observation. It is important to bear in mind the possible bias in these responses since respondents may have given a more favorable perspective of their facilities, leading to an underestimation of the gaps. At the same time, it can also be difficult to judge if respondents tended to exaggerate the gaps in their respective facilities to attract attention. Finally, this was a cross-sectional study, meaning that causal effects could not be inferred and only associations were reported. Despite these limitations, the findings of this study add valuable insights to the growing body of knowledge, revealing important gaps and how a fragmented approach can frustrate or slow down the progress towards improving NCDs services at all levels irrespective of existing health systems structures. This evidence could be used to help improve the management of NCDs in low-resource settings.

## Conclusions

Shortcomings in the current readiness of facilities to manage NCDs in Kenya at different levels of health care were revealed, showing variations by disease and type of healthcare facility. It is, therefore, critical to invest more in strengthening existing health systems to provide low-cost but effective NCD interventions through integrated efforts and prioritization of disease burden in the population at all levels as part of routine health care. Other countries in LMICs can also draw lessons from this study. By enhancing the ability of health facilities to manage different NCDs concurrently would help to minimize the burden of NCD-related disability and premature deaths in these limited resources settings. In conclusion, to bridge the gap between population and health care needs for NCD management and prevention, more concerted efforts are required in delivering a more “complete-package” approach across all service-specific domains for the different NCDs.

## Supplementary Information

**Additional file 1.** Facility Assessment Questionnaire for Noncommunicable Diseases Management.

**Additional file 2.** Tracer indicator items for non-communicable disease service readiness**.**

## Data Availability

The datasets used in the current study are not publicly available due to confidentiality of some of the data but can be accessed following a reasonable request to the African Population and Health Research Center (APHRC) through its Microdata portal (https://aphrc.org/microdata-portal/).

## Abbreviations

CRD: Chronic respiratory disease
CVD: Cardiovascular diseases
DALYs: Disability-adjusted life years
LMICs: Low- and middle-income countries
NCDs: Non-communicable diseases
ORs: Odds ratios
SARA: Service availability and readiness assessment
SSA: Sub-saharan africa
WHO: World health organization
WHO-PEN: WHO package of essential non-communicable diseases
