# Supplementary material for: Readiness of health facilities to deliver non-communicable diseases services in Kenya: a national cross-sectional survey
Source: BMC Health Serv Res. 2022 Aug 2;22:985. doi: 10.1186/s12913-022-08364-w (PMC9344761; doi:10.1186/s12913-022-08364-w)
Supplement: Supplementary file 1 — Additional file 1. Facility Assessment Questionnaire for Noncommunicable Diseases Management. [file 12913_2022_8364_MOESM1_ESM.pdf]

Additional File 1: Facility Assessment Questionnaire for Noncommunicable Diseases Management

African Population & Health Research Center

Facility Assessment Questionnaire

County: ______________

Interviewer: _________________

Data collection date: | d | d | | m | m | m | | y | y | y | y |

Facility name: ____

Facility setting type: ☐1 Public ☐2 Private for profit ☐3 Private-faith based

Facility Level ☐1 level II ☐2 Level III ☐3 Level IV☐4 Level V ☐5 level VI

GPS Info: _________________/____________________
[Latitude] [Longitude]

A. General Information

This facility serves: ______________________/______________________/______________________/___________________
[County] [sub-county] [Location] [sub-location]

How many primary health care facilities serve this area? __________ **public** facilities __________**mission/private facilities**

How many people this facility serves? _________ households _________ people

B. Equipment & Service Availability

Availability of basic equipment for chronic diseases (CDs) management (number of **functional** devices available)

***Note:** *fill in "0" if there is none (this note applies to all the following questions involved with numbers)*

Equipment	Weighing machines	Measuring tape	Stethoscope	Glucometer	ECG machines
Number	________	________	________	________	________

Availability of Blood Pressure Measuring Devices (BPMDs):

Type	Number	How often are BPMDs calibrated for accuracy?
Mercury	________	☐1Once a year or more ☐2Less than once a year ☐3Never ☐4Don't know
Aneroid	________	☐1Once a year or more ☐2Less than once a year ☐3Never ☐4Don't know
Automatic (non-portable)	________	☐1Once a year or more ☐2Less than once a year ☐3Never ☐4Don't know
Automatic (portable & wrist cuff)	________	☐1Once a year or more ☐2Less than once a year ☐3Never ☐4Don't know
Automatic (portable & upper arm)	________	☐1Once a year or more ☐2Less than once a year ☐3Never ☐4Don't know

Availability of medical procedures and laboratory tests:

Injection	IV fluid	Blood sugar test	Blood cholesterol test
☐1Yes ☐2No	☐1Yes ☐2No	Finger tip ☐1Yes ☐2No<br>Venipuncture ☐1Yes ☐2No	Fingertip ☐1Yes ☐2No<br>Venipuncture ☐1Yes ☐2No
Pap smear for cancer screening	Urine protein dipstick testing	Urine ketone dipstick testing	
☐1Yes ☐2No	☐1Yes ☐2No	☐1Yes ☐2No	

**I would like to know if the following items for diagnostic testing are available or not available today**	OBSERVED AVAILABLE			NOT OBSERVED		
	OBSERVED	REPORTED NOT SEEN	NOT AVAILABLE	YES	NO	DON'T KNOW
Dipstick for urine protein	1	2	3	1	2	3
Dipstick for urine glucose	1	2	3	1	2	3
Dipstick for urine ketone bodies	1	2	3	1	2	3
X-ray machine	1	2	3	1	2	3
Ultrasound equipment	1	2	3	1	2	3
CT scan	1	2	3	1	2	3
ECG	1	2	3	1	2	3

C. Availability of NCD-specific services

Cervical Cancer

Do providers in this facility diagnose and/manage cervical cancer?	☐1Yes ☐2No
Do you have a national guideline for diagnosis and management of cervical cancer available in this facility? If available ask to see the document	☐1 Yes, observed ☐2 Yes, reported, not seen ☐3No
Have you or nay provider (s) received any training in the diagnosis and management of cervical cancer?	☐1Yes ☐2No
Please tell if the following basic equipment are available in this service area today?	

	A) AVAILABLE			B) FUNCTIONING		
	OBSERVED	REPORTED NOT SEEN	NOT AVAILABLE	YES	NO	DON'T KNOW
Acetic acid	1	2	3	1	2	3
Speculum	1	2	3	1	2	3

Diabetes

Do providers in this facility diagnose and/manage diabetes?	☐1Yes ☐2No
Do you have a national guideline for diagnosis and management of diabetes available in this facility? If available ask to see the document	☐1 Yes, observed ☐2 Yes, reported, not seen ☐3No

Have you or nay provider (s) received any training in the diagnosis and management of diabetes?	☐$_1$Yes ☐$_2$No

Cardiovascular disease (CVD)

Do providers in this facility diagnose and/manage CVD?	☐$_1$Yes ☐$_2$No
Do you have a national guideline for diagnosis and management of CVD available in this facility? If available ask to see the document	☐$_1$ Yes, observed ☐$_2$ Yes, reported, not seen ☐$_3$No
Have you or nay provider (s) received any training in the diagnosis and management of CVD?	☐$_1$Yes ☐$_2$No

Chronic respiratory disease (CRD)

Do providers in this facility diagnose and/manage chronic respiratory diseases?	☐$_1$Yes ☐$_2$No	
Do you have a national guideline for diagnosis and management of chronic respiratory disease available in this facility? If available ask to see the document	☐$_1$ Yes, observed ☐$_2$ Yes, reported, not seen ☐$_3$No	
Have you or nay provider (s) received any training in the diagnosis and management of chronic respiratory diseases?	☐$_1$Yes ☐$_2$No	
	A) AVAILABLE	B) FUNCTIONING

Please tell if the following basic equipment are available in this service area today?	OBSERVED	REPORTED NOT SEEN	NOT AVAILABLE	YES	NO	DON'T KNOW
Peak flow meters	1	2	3	1	2	3
Spacers for inhalers	1	2	3	1	2	3

D. Medicine

**Medicine**	**Availability**	**Prescription right**
Acetylsalicylic acid (aspirin)	☐1Always ☐2Sometimes ☐3No	☐1Yes ☐2No
Angiotensin inhibitor (enalapril)	☐1Always ☐2Sometimes ☐3No	☐1Yes ☐2No
Beta-blocker (atenolol)	☐1Always ☐2Sometimes ☐3No	☐1Yes ☐2No
Calcium channel blocker Now (amlodipine)	☐1Always ☐2Sometimes ☐3No	☐1Yes ☐2No
Epinephrine	☐1Always ☐2Sometimes ☐3No	☐1Yes ☐2No
Furosemide	☐1Always ☐2Sometimes ☐3No	☐1Yes ☐2No
Glyceryl trinitrate	☐1Always ☐2Sometimes ☐3No	☐1Yes ☐2No
Heparin	☐1Always ☐2Sometimes ☐3No	☐1Yes ☐2No
Isosorbide dinitrate	☐1Always ☐2Sometimes ☐3No	☐1Yes ☐2No
Spironolactone	☐1Always ☐2Sometimes ☐3No	☐1Yes ☐2No
Statin (simvastatin)	☐1Always ☐2Sometimes ☐3No	☐1Yes ☐2No
Thiazide diuretic	☐1Always ☐2Sometimes ☐3No	☐1Yes ☐2No
Insulin	☐1Always ☐2Sometimes ☐3No	☐1Yes ☐2No
Metformin	☐1Always ☐2Sometimes ☐3No	☐1Yes ☐2No
Glibenclamide	☐1Always ☐2Sometimes ☐3No	☐1Yes ☐2No
Verapamil	☐1Always ☐2Sometimes ☐3No	☐1Yes ☐2No

Digoxin	☐1Always ☐2Sometimes ☐3No	☐1Yes ☐2No
Lignocaine	☐1Always ☐2Sometimes ☐3No	☐1Yes ☐2No
Hydralazine	☐1Always ☐2Sometimes ☐3No	☐1Yes ☐2No
Hydrochlorothiazide	☐1Always ☐2Sometimes ☐3No	☐1Yes ☐2No
Methyldopa	☐1Always ☐2Sometimes ☐3No	☐1Yes ☐2No

Supplement List

Amiodarone	☐1Always ☐2Sometimes ☐3No	☐1Yes ☐2No
Sodium nitroprusside	☐1Always ☐2Sometimes ☐3No	☐1Yes ☐2No
Dopamine	☐1Always ☐2Sometimes ☐3No	☐1Yes ☐2No
Streptokinase	☐1Always ☐2Sometimes ☐3No	☐1Yes ☐2No

That's end. Thanks for your participation!
