# Supplementary material for: Readiness of health facilities to deliver non-communicable diseases services in Kenya: a national cross-sectional survey
Source: BMC Health Serv Res. 2022 Aug 2;22:985. doi: 10.1186/s12913-022-08364-w (PMC9344761; doi:10.1186/s12913-022-08364-w)
Supplement: Supplementary file 2 — Additional file 2. Tracer indicator items for non-communicable disease service readiness. [file 12913_2022_8364_MOESM2_ESM.docx]

# **Supplementary Information**

**Additional file 2: Tracer indicator items for non-communicable disease service readiness.**

| NCD-specific service | Domain | Indicator tracer item |
| --- | --- | --- |
| Diabetes readiness | Trained staff and guidelines | - Guidelines for diabetes diagnosis and treatment - Staff trained for diabetes diagnosis and treatment |
|  | Equipment | - Blood pressure apparatus - Weighing machine - Measuring tape - Blood glucose test - Urine dipstick-protein - Urine dipstick-ketones |
|  | Medicines and commodities | - Metformin capsules/tablets - Glibenclamide capsules/tablets - Insulin regular injectable - Gliclazide tablet or glipizide tablet |
| Cardiovascular disease (CVD) readiness | Trained staff and guidelines | - Guidelines for diagnosis and treatment of CVD - Staff trained in diagnosis and management of CVD |
|  | Equipment | - Stethoscope - Blood pressure apparatus - Weighing machine |
|  | Medicines and commodities | - ACE inhibitor (enalapril) - Thiazide - Beta-blocker (atenolol) - Calcium channel blocker (amlodipine) - Aspirin (acetylsalicylic acid) capsules/tablets - Metformin capsules/tablets - Hydrochlorothiazide tablet or other thiazide diuretic tablet |
| Chronic respiratory disease (CRD) readiness | Trained staff and guidelines | - Guidelines for diagnosis and management of CRD - Staff trained in diagnosis and management of CRD |
|  | Equipment | - Stethoscope - Peak flow meter - Spacers for inhalers |
|  | Medicines and commodities | - Epinephrine injectable |
| Cervical cancer screening readiness | Trained staff and guidelines | - Guidelines for cervical cancer prevention and control - Staff trained in cervical cancer prevention and control |
|  | Equipment | - Speculum |
|  | Diagnostics | - Acetic acid |
